# Effect of Ion Channel Randomness on Sensitivity of Neurons to External Electromagnetic Fields: Computational Study

**DOI:** 10.3390/e28060581

**Published:** 2026-05-22

**Authors:** Arkady Pikovsky, Andreas Deser

**Affiliations:** 1 Department of Physics and Astronomy, University of Potsdam, Karl-Liebknecht-Str. 24/25, 14476 Potsdam-Golm, Germany; 2 Unatech GmbH, An der Kollonade 11, 10117 Berlin, Germany; 3 German Federal Office for Radiation Protection, Competence Center for Electromagnetic Fields, Ingolstädter Landstraße 1, 85764 Oberschleißheim, Germany; adeser@bfs.de

**Keywords:** spiking neuron, periodic driving, ion channel stochasticity

## Abstract

We perform stochastic simulations of the Hodgkin–Huxley and Morris–Lecar models with different numbers of ion channels in order to describe the effects of periodic electrical driving on spike rates and the regularity of spiking in a single neuron. For stochastic modeling, we use an efficient method that reduces the piecewise-deterministic Markov process of the membrane potential evolution to an ordinary differential equation between random opening and closing events. To characterize a regular component in the resulting voltage time series, we adopt a Wiener order parameter based on the autocorrelation function. We show that the effect of ion channel stochasticity on the spike rate is stronger at lower external force frequencies. The regular component of neural activity exhibits resonant-like behavior as a function of the driving frequency, with a maximum in the beta range.

## 1. Introduction

Information transfer in the nervous system is mediated by electrically excitable cells called neurons. As in all cells, differences in ion concentrations inside and outside the cell give rise to an electrical potential across the cell membrane, called the transmembrane potential. Along with other means, ion transport in neurons occurs through voltage-gated ion channels, in which the probability of a conformational change between the open and closed state depends on the membrane potential [1,2]. When the latter reaches a certain threshold, this can trigger transient spikes in membrane voltage called action potentials, which propagate along the neuron.

In the simplest conductance-based point neuron models, the dynamics of the membrane potential are described by a set of ordinary differential equations for the voltage and the fractions of the relevant ion channels in the open state. The archetypal and widely used set of equations of this type is the Hodgkin–Huxley model, for which A.L. Hodgkin and A.F. Huxley, together with J. C. Eccles, were awarded the Nobel Prize in Physiology or Medicine [3].

A refinement of such models consists in treating the random opening and closing of ion channels, and hence the fraction of open channels, as a time-continuous Markov process [4,5] or as a piecewise deterministic Markov process (PDMP) [6,7,8]. It can be shown that, in the limit of a large number of ion channels, stochastic models of the transmembrane potential converge to the classical deterministic models [9,10,11]. Interpreting these results as laws of large numbers, the central limit theorem corresponds to approximating these Markov processes by diffusion processes [12,13] and similarly for the piecewise deterministic Markov case [6,7,14]. Following this line of reasoning, the third step is large deviation theory, corresponding to the occurrence of spontaneous action potentials and their rate in the resting state of a neuron [15,16,17]. It is often assumed that simulations of stochastic differential equations resulting from the diffusion approximations are more efficient than direct Monte Carlo simulations of the Markov processes [18,19].

A refinement along a different line is to explicitly consider the spatial structure of a neuron in a simplified way. A class of theories that implements this idea consists of those models termed “spatially extended”, in which the axon is partitioned into compartments by the nodes of Ranvier. This leads to systems of coupled ordinary or stochastic differential equations for the membrane potentials in each compartment [20,21,22].

Due to the inherently electromagnetic nature of action potential generation, it is evident that electromagnetic fields (EMF) and currents induced in tissue may act as external driving forces, meaning that they can influence neural excitation. Thus, safety guidelines aim to protect against EMF-induced low-frequency fields and currents.

The International Commission on Non-Ionizing Radiation Protection (ICNIRP) develops and publishes guidelines for limiting exposure to low-frequency electric and magnetic fields (see [23]; according to the present workplan, an update is in progress [24]). These are based on the current state of scientific knowledge regarding the potential effects of such fields on human health. In the frequency range from 1 Hz up to 100 kHz, the primary biological effect of electric and magnetic fields is the stimulation of muscles and neurons in the peripheral and central nervous systems. Neuronal stimulation occurs through a change in the transmembrane potential induced by a locally applied electric field. Hence, tissue internal electric fields should not exceed values that would cause a crossing of firing thresholds. The latter is the physical quantity for which exposure limits are defined in safety regulations. The relationship between the local electric field strength and the driving term in the transmembrane potential equations depends on the dielectric tissue parameters, the geometry of the neuron, and other coupling factors [22,25]. It is based on the relation J=σE, where *J* is the locally-induced current density and σ is the electrical conductivity tensor. Integrating *J* over the relevant volume of neural tissue yields a current $I_{ext}$ that serves as a driving term in the dynamical model for the action potential.

The main computational protocol currently employed in the numerical determination of action potential thresholds is based on the *spatially extended nonlinear node* (SENN) model [20,21,25,26]. It describes a myelinated neuron, insulated from the extracellular medium by a lipid layer (myelin) but with Ranvier nodes where the lipid is absent. This results in a system of coupled ordinary differential equations, with the dynamics at each node described by the Frankenhäuser–Huxley equations [27]. The protocol for determining the action potential thresholds is as follows: first, fix the geometry of the external currents relative to the chain of nodes and choose a form of the external field (e.g., sinusoidal, pulsed, etc.); then, for a given frequency, increase the amplitude of the external current and find the threshold at which spiking occurs. This amplitude is the critical amplitude.

An assessment of the conservativeness of action potential thresholds obtained via Frankenhäuser–Huxley dynamics and comparison to other dynamical models was performed recently [28]. It was observed that limit values strongly depend on the underlying membrane dynamics. In the present article, we emphasize another shortcoming of these types of neuron models. In the SENN protocol, the underlying dynamics are deterministic, i.e., any effects due to the stochastic nature of the ion channel dynamics in neurons are neglected. The presence of spontaneous action potentials in the resting state of a neuron [15] indicates already that firing thresholds and even more, the definition of a firing threshold appears to be different in more realistic models, based on stochastic membrane dynamics.

The goal of this paper is to explore the effects of ion channel stochasticity on the neural dynamics under external electromagnetic fields. We investigate the Hodgkin–Huxley and Morris–Lecar models. The first challenge is a time-efficient simulation method which does not contain uncontrolled approximations. Here, we follow a recent publication [29] in which such a method was suggested and demonstrate its applicability to forced stochastic neural dynamics. We consider two particular models of different complexity for which properties of stochastic ion channel dynamics are well established: the classical Hodgkin–Huxley (HH) model, and the Morris–Lecar (ML) model. We are not aware of an ion-channel-based stochastic formulation of the Frankenhäuser–Huxley model; therefore, it is not considered below. We introduce the HH and ML models in Section 2.1. We describe the efficient simulation method in Section 2.2. One of the central issues in extending SENN models to the stochastic case is the absence of a sharp transition to spiking, as observed in the deterministic case. In the presence of ion channel stochasticity, spontaneous irregular spikes occur in a stable excitable regime, and it is a priori not clear how to define a value for the electric field leading to a firing threshold in this case. In this respect, it is also interesting to explore the ordering effect of an external periodic forcing on random spontaneous spiking. Indeed, a significant periodic component in spiking activity can potentially influence the ability of neural networks to process information. Therefore, it is important not only to model spike rates in the presence of periodic forces but also to detect the presence of a periodic component in the spiking activity. We argue in Section 2.3 that this can be accomplished with a Wiener order parameter, which can be straightforwardly calculated from the autocorrelation function of the voltage signal. In Section 3, we apply the methods outlined in Section 2 to the Hodgkin–Huxley and Morris–Lecar models. We show that spike rates and periodic components in the voltage activity can be reliably calculated across a wide range of system sizes (number of ion channels involved) and forcing parameters (amplitude and frequency). Finally, in Section 4 we discuss limitations of the approach and possible extensions.

## 2. Models and Methods

### 2.1. Hodgkin–Huxley and Morris–Lecar Models of Neural Activity

Here, we introduce the Hodgkin–Huxley (HH) (see review in [30]) and Morris–Lecar (ML) ([31], see review in [32]) models that we explore below and give their formulation as random processes due to stochastic openings and closing of ion channels [1].

#### 2.1.1. Deterministic and Stochastic HH Model

The deterministic HH model is an equation for the neuronal membrane voltage evolution derived from electric charge conservation, i.e., Kirchhoff’s law for the currents entering and leaving the cell:

(1) $$C\frac{dV}{dt}+I_{ion}=I_{ext},\;I_{ion}=I_{Na}+I_{K}+I_{l}\;,$$

where *V* is the membrane voltage in millivolts, *C* represents the membrane capacitance (usually set to 1 mF cm^−2^), and $I_{ext}$ is the stimulus current added to the neuron in mA cm^−2^. The values of $I_{Na}$, $I_{K}$, and $I_{l}$ are the currents of the Na+, K+, and leakage channels, respectively, given by

(2) $$I_{Na}=g_{Na}\rho_{Na}\left(V-V_{Na}\right),\;I_{K}=g_{K}\rho_{K}\left(V-V_{K}\right),\;I_{l}=g_{l}\left(V-V_{l}\right)\;.$$

The values $\rho_{Na}$ and $\rho_{K}$ respectively denote the open fractions of the Na+ and K+ channels. $V_{Na}$ and $V_{K}$ are the Nernst potentials of the corresponding channels and $V_{l}$ is the leakage potential, which is measured via voltage clamp experiments and the value of which is given below. In the HH model, each Na+ channel contains two types of subunits, including three type-*m* subunits and one type-*h* subunit. Each K+ channel is composed of four type-*n* subunits. If all four subunits are in the activation (open) state, then the Na+ or K+ channel is defined as open. In the deterministic limit, one sets $\rho_{Na}=m^{3}h$ and $\rho_{K}=n^{4}$. The equations for the activation state variables n,m,h read

(3) $$\begin{array}{cc} \frac{dn}{dt} & =\alpha_{n}\left(1-n\right)-\beta_{n}n\;,\\ \frac{dm}{dt} & =\alpha_{m}\left(1-m\right)-\beta_{m}m\;,\\ \frac{dh}{dt} & =\alpha_{h}\left(1-h\right)-\beta_{h}h\;. \end{array}$$

Dependencies of the factors α and β on the voltages have been determined experimentally. Here, we write the full standard HH model with the parameter values used below (according to the book [33], Section 2.3.1):

(4) $$\begin{array}{cc} C\frac{dV}{dt} & =I_{ext}-{\bar{g}}_{Na}m^{3}h\left(V-V_{Na}\right)-{\bar{g}}_{K}n^{4}\left(V-V_{K}\right)-g_{l}\left(V-V_{l}\right)\;,\\ \frac{dn}{dt} & =\alpha_{n}\left(1-n\right)-\beta_{n}n,\;\alpha_{n}=0.01\frac{-V+10}{\exp[\frac{-V+10}{10}]-1},\;\beta_{n}=0.125\exp\left[-V/80\right],\\ \frac{dm}{dt} & =\alpha_{m}\left(1-m\right)-\beta_{m}m,\;\alpha_{m}=0.1\frac{-V+25}{\exp[\frac{-V+25}{10}]-1},\;\beta_{m}=4\exp\left[-V/18\right],\\ \frac{dh}{dt} & =\alpha_{h}\left(1-h\right)-\beta_{h}h,\;\alpha_{h}=0.07\exp\left[-V/20\right],\;\beta_{h}=\frac{1}{\exp[\frac{-V+30}{10}]+1}\;. \end{array}$$

In order to facilitate comparisons to experimental measurements, scales of the observables in the model have to be specified. In the HH model written above, voltage *V* is measured in mV, current $I_{ext}$ in μA/cm^2^, and time *t* in ms. Other parameter values are as follows:

$$\begin{array}{c} C=1\;\mu {F/\mathrm{cm}}^{2},\;V_{Na}=120\;\mathrm{mV},\;V_{K}=-12\;\mathrm{mV},\;V_{l}=10.6\;\mathrm{mV},\\ {\bar{g}}_{Na}=120\;{\mathrm{mS}/\mathrm{cm}}^{2},\;{\bar{g}}_{K}=36\;{\mathrm{mS}/\mathrm{cm}}^{2},\;{\bar{g}}_{l}=0.3\;{\mathrm{mS}/\mathrm{cm}}^{2}. \end{array}$$

In the HH model, the currents are not proportional to the activation variables but to their powers $\rho_{Na}=m^{3}h$ and $\rho_{K}=n^{4}$, which give the portion of open channels. This is because channels are composite entities consisting of several subunits.

In the stochastic channel-based formulation [1,34], a potassium channel (we denote the total number of potassium channels by *N*) consists of four subunits, and is open only if all four subunits are open. Diagram (5) shows how to proceed to the channel-state formulation, starting from the subunit formulation, under the assumption that all sub-units are independent and kinetically identical. Each channel has five states, depending on how many of its subunits are open (from $n_{0}$, where all subunits are closed, to $n_{4}$ where all are open); only one of these states (denoted n4 in (5)) is the state where the whole channel is open.

(5) n0⇄βn4αnn1⇄2βn3αnn2⇄3βn2αnn3⇄4βnαnn4

Diagram (5) should be interpreted as follows. Because each channel can be in one of five states, the sum $n_{0}+n_{1}+n_{2}+n_{3}+n_{4}=N$ is the total number of potassium channels; the transition rates are proportional to the numbers of the corresponding subunits. For example, in state n1 each channel has three closed subunits and one open unit. Therefore, the transition to n0 happens with the closing rate for one subunit, i.e., with rate $\beta_{n}$. The transition to n2 occurs if one of the three closed subunits opens; the rate for this is the opening rate for one subunit multiplied by 3, i.e., $3\alpha_{n}$. In the deterministic limit of a very large number of channels, if the probability of a subunit being open is *n*, then the probability of state n4 (where all subunits are open) is $n^{4}$; thus, $\rho_{K}\propto n^{4}$ for the potassium current in (2).

A similar picture for the Na channel (with the total number of sodium channels denoted by *M*) includes four states for the variable *m* and two states for the variable *h*; altogether, each Na channel can be in eight different states. We show this representation in Diagram (6) where, the open state is m3h0.

(6) m0h0⇄βm3αmm1h0⇄2βm2αmm2h0⇄3βmαmm3h0βh↑↓αhβh↑↓αhβh↑↓αhβh↑↓αhm0h1⇄βm3αmm1h1⇄2βm2αmm2h1⇄3βmαmm3h1

The interpretation of this diagram is the same as of Diagram (5) above.

#### 2.1.2. Deterministic and Stochastic ML Model

Probably the simplest nontrivial model of neuronal spiking that allows for a stochastic representation is the Morris–Lecar (ML) model [31]. The advantage of the ML model is that it can be easily implemented on all levels: (i) in the deterministic limit; (ii) in an exact simulation for a finite number of ion channels using the Monte Carlo algorithm; and (iii) in the diffusion approximation, where the irregularity of ion channels is modeled using a Langevin equation (see, e.g., [35,36]).

Here, in contrast to the HH and FH models, only potassium channels (variable *n*) are treated as dynamical units. In addition, there are calcium channels, which are considered as fast channels, so that the activation variable *m* instantaneously takes its equilibrium value $m_{\infty }\left(V\right)$ at a given voltage. The deterministic ML equations read as follows:

(7) $$\begin{array}{cc} \frac{dV}{dt} & =F\left(V,n\right)=\frac{1}{C}\left(I_{ext}-g_{Ca}m_{\infty }\left(V-V_{Ca}\right)-g_{L}\left(V-V_{L}\right)-g_{K}n\left(V-V_{K}\right)\right),\\ \frac{dn}{dt} & =(1-n)\alpha (V)-n\beta (V), \end{array}$$

where the fraction of open potassium channels $n=N_{open}/N$ is considered as a continuous variable with *N* as the total number of potassium channels and where

(8) $$\begin{array}{c} m_{\infty }=\frac{1}{2}\left(1+\tanh\left(\frac{V-V_{a}}{V_{b}}\right)\right),\\ \alpha \left(V\right)=\phi \frac{\cosh(\xi /2)}{1+e^{-2\xi }},\;\beta \left(V\right)=\phi \frac{\cosh(\xi /2)}{1+e^{2\xi }},\;\xi =\frac{V-V_{c}}{V_{d}}. \end{array}$$

This equation can be rewritten as

(9) $$\begin{array}{cc} \frac{dV}{dt} & =F\left(V,n\right)=\frac{1}{C}\left(I_{ext}-g_{Ca}m_{\infty }\left(V-V_{Ca}\right)-g_{L}\left(V-V_{L}\right)-g_{K}n\left(V-V_{K}\right)\right)\;\\ \frac{dn}{dt} & =\frac{n_{\infty }-n}{\tau },\\ \; & n_{\infty }=\frac{1+\tanh\xi }{2}=\frac{\alpha }{\alpha +\beta },\;\tau =\frac{1}{\phi \cosh(\xi /2)}=\frac{1}{\alpha +\beta }. \end{array}$$

The parameters adopted in [35] and henceforth in this paper are

$$\begin{array}{c} C=20,\;V_{K}=-84,\;V_{L}=-60,\;V_{Ca}=120,\\ I_{ext}=100,\;g_{K}=8,\;g_{L}=2,\;g_{Ca}=4.4\;\\ V_{a}=-1.2,\;V_{b}=18,\;V_{c}=2\;\;V_{d}=30,\;\phi =0.04. \end{array}$$

In the deterministic version, one considers the variable n(t) denoting the fraction of open potassium channels as a continuous variable with 0≤n≤1. In the stochastic setting, the number of channels *N* is finite and the number of open channels is an integer $0\leq N_{open}\leq N$; accordingly, $n=N_{open}/N$ takes only a finite set of values. In the stochastic formulation, potassium channels are considered as random units with two states, open (1) and closed (0):

0⇄βα1

where the rates α,β depend on the voltage *V* as presented in Equation (8). Because all the channels are identical, if one has $N_{open}$ open channels at some time instant, then the rate for opening one more channel (i.e., of the transition $N_{open}\to N_{open}+1$) is $\alpha (N-N_{open})$, and the rate of closing a channel (i.e., of the transition $N_{open}\to N_{open}-1$) is $\beta N_{open}$. Thus, the Markovian process of openings and closings can be formulated as

(10) $$N_{open}\to \left\{\begin{array}{cc} N_{open}+1 & \mathrm{rate}\;\alpha \left(V\right)\left(N-N_{open}\right)\;,\\ N_{open}-1 & \mathrm{rate}\;\beta \left(V\right)N_{open}\;. \end{array}\right.$$

The deterministic part of the dynamics reads

(11) $$\frac{dV}{dt}=F\left(V,n\right)=\frac{1}{C}\left(I_{ext}-g_{Ca}m_{\infty }\left(V-V_{Ca}\right)-g_{L}\left(V-V_{L}\right)-g_{K}\frac{N_{open}}{N}\left(V-V_{K}\right)\right).$$

The included functions are given by the expressions in (8).

#### 2.1.3. Forcing Term

To model the effect of external electromagnetic fields on a neuron, a periodic component is added to the external current:

(12) $$I_{ext}\to I_{ext}+A\cos\left(2\pi ft\right)\;.$$

The amplitude *A* and frequency *f* are the parameters of the force. We note here that more complex waveforms of the forcing field have also been studied in the literature, e.g., two-frequency forcing was explored by [37].

### 2.2. Numerical Simulation of Piece-Wise Deterministic Markov Processes

From a mathematical viewpoint, the neural stochastic models discussed above in Section 2.1 are piecewise-deterministic Markov processes (PDMP), which constitute a broad class of stochastic processes with many applications. Mathematical foundations and properties can be found in [36,38,39,40,41]. Sometimes, PDMPs are called hybrid stochastic systems [8]. Roughly speaking, a PDMP is a generalization of a standard Markov process consisting of jumps at random times to a situation in which some variables also evolve deterministically between jumps. A classic example of a PDMP is provided by the stochastic neuron dynamics introduced in Section 2.1. The membrane voltage V(t) is a continuous variable that varies deterministically according to the capacitance discharge equations (Equations (1) and (11)). The conductances of ion channels are random because they can spontaneously open and close, which is modeled using Markov processes. The random and deterministic dynamics depend on each other: the voltage discharge depends on random conductances, and the rates at which the channels open and close depend on the voltage.

Here, we formulate a rather generic PDMP. The dynamics consist of purely deterministic evolution epochs interrupted by discrete jump events. We have a set of variables $\vec{X}\left(t\right)$ that evolves during deterministic epochs according to the following ODE:

(13) $$\frac{d\vec{X}}{dt}=\vec{F}\left(\vec{X},\vec{Y},t\right)\;.$$

There may exist another set of variables $\vec{Y}$ which varies only at jump events and remains constant during deterministic evolution (13). Variables $\vec{X}$ can generally also vary at jump events. The variables $\vec{Y}$ are discrete, while the variables $\vec{X}$ can be continuous or discrete. For simplicity, in what follows we call the variables $\vec{X}$ “continuous” and the variables $\vec{Y}$ “discrete”.

There are generally *L* different types of discrete events, which are assumed to all be independent Markov processes with rates

(14) $$\lambda_{i}\left(\vec{X},\vec{Y},t\right),\;i=1,\ldots ,L\;,$$

that is, an event *i* occurs within a small time interval (t,t+dt) with probability $\lambda_{i}\left(\vec{X}\left(t\right),\vec{Y}\left(t\right),t\right)\;dt$. For example, in the context of the stochastic formulation of the neural models above, discrete events are openings and closings of subunits of ion channels. In Diagrams (5) and (6), the number of events is the number of arrows. Thus, for the HH model, L=28 (eight possible transitions in (5) and twenty possible transitions in (6)), while for the ML model Equation (10) gives L=2.

Generally, if an event happens, then all dynamical variables $\vec{X},\vec{Y}$ are transformed according to deterministic or probabilistic rules. However, in some applications only discrete variables vary at the jumps. We will assume that these transformations can be easily implemented in numerical simulations. Provided that the r.h.s. is smooth enough, the evolution problem in (13) between the jumps reduces to a standard numerical task of solving a system of ordinary differential equations. Usually, this is accomplished using a variant of the Runge–Kutta method. The main challenge in numerical simulations is modeling the discrete jump times.

In the context of stochastic neuronal dynamics as described in Section 2.1, there is one continuous variable V(t). The number of discrete variables (open and closed channels or their subunits) differs across models.

#### 2.2.1. Classical Gillespie Direct Method (GDM)

D.T. Gillespie developed three methods for efficient simulation of the Markov processes [42,43,44], and see recent reviews in [45,46]. Here, we succinctly describe the Gillespie direct method (GDM), which is applicable in the simplest case of constant (between the transitions) rates, i.e., of the rates $\lambda_{i}\left(\vec{Y}\right)$ that do not depend on the continuous variables $\vec{X}\left(t\right)$ and time *t*.

The Gillespie method is based on the following properties of Markov processes (see, e.g., [47]). The waiting time for a Markov process with rate λ has distribution density $\psi \left(\tau \right)=\lambda e^{-\lambda \tau }$. A superposition of Markov processes with rates $\lambda_{i}$ is a Markov process with rate $\Lambda =\sum_{i}\lambda_{i}$. If an event is generated according to the superposition, the probability $\Pi_{i}$ for a process *i* to occur is $\Pi_{i}=\lambda_{i}/\Lambda$. The Gillespie algorithm described below assumes that the reaction rates remain constant between the events.

0. Initialization:
  (a) Define the system’s initial state and set t=0;
  (b) Calculate the rate $\lambda_{j}$ for each reaction channel *j*;
  (c) Calculate the total rate $\Lambda =\sum_{j=1}^{L}\lambda_{j}$.
1. Draw a random variate $u_{1}$ from a uniform distribution on (0,1] and generate the waiting time by $\tau =-\ln u_{1}/\Lambda$.
2. Draw $u_{2}$ from a uniform distribution on (0,Λ]. Select the event *i* to occur by iterating over i=1,2,…,L until finding that *i* for which $\sum_{j=1}^{i-1}\lambda_{j}<u_{2}\leq \sum_{j=1}^{i}\lambda_{j}$.
3. Perform the event on reaction channel *i*.
4. Advance the time according to t→t+τ.
5. Update $\lambda_{i}$ as well as all other $\lambda_{j}$ and Λ that are affected by the produced event.
6. Return to Step 1.

The essence of the GDM algorithm is in Steps 1 and 2; in Step 1, the time interval to the next event is calculated as a sample of an exponentially distributed random number with time constant Λ, and in Step 2 the type of event (one out of *L* possible types) is determined by sampling a discrete distribution with probabilities $\lambda_{i}/\Lambda$. GDM requires two random number generations per step. In [45], several ways are described to accelerate the GDM. The GDM in this form has been adopted in many simulations of the stochastic HH model [15,18,19,48,49,50] by assuming weak dependence of rates on the voltage V(t) and the time. In [19], it is mentioned that using piecewise-constant rates (i.e., neglecting the voltage-dependence of these rates on the time intervals between discrete events) gives statistics similar to the exact simulation if the number of channels is larger than 40. However, they also mention that no detailed comparison has been performed. Figures 4 and 5 of [35] indicate that the differences between the exact and piecewise-constant algorithms for the ML model are indeed minor for channel numbers larger or equal to 40.

#### 2.2.2. Approximate vs. Exact Simulation of Jump Times in the GDM

For time-dependent rates $\lambda_{i}\left(t\right)$, the usual GDM is amended as follows. The survival function Ψ(τ;t) is defined as the probability of not having an event in the time window [t,t+τ]. It is the product of the probabilities not having an event in small time intervals (altogether *r* intervals), and can be reformulated as an integral

$$\Psi_{i}\left(\tau ;t\right)\approx \prod_{q=0}^{r-1}\left[1-\lambda_{i}\left(t+q\frac{\tau }{r}\right)\frac{\tau }{r}\right]=\exp\left(-\int_{t}^{t+\tau }\lambda_{i}\left(s\right)ds\right)\;.$$

Now, consider *M* parallel independent processes. If the time of the last event was $t^{last}$, then the total survival function is the product

(15) $$\Psi \left(\tau ;t^{last}\right)=\prod_{i=1}^{M}\Psi_{i}\left(\tau ;t^{last}\right)=\exp\left(-\int_{t^{last}}^{t^{last}+\tau }\Lambda \left(s\right)ds\right)\;,$$

where as above $\Lambda \left(t\right)=\sum_{i}\lambda_{i}\left(t\right)$.

It is appropriate to introduce the cumulative rate according to

(16) $$\Phi \left(\tau \right)=\int_{t^{last}}^{t^{last}+\tau }\Lambda \left(s\right)ds\;.$$

According to Equation (15), this quantity follows an exponential distribution with unit time; thus, one attributes Φ=−lnu, where *u* is uniformly distributed in (0,1], then finds τ from Equation (16) so that $t^{new}=t^{last}+\tau$.

Alternatively, one can write an ODE for Φ:

(17) $$\frac{d\Phi }{dt}=\Lambda \left(t\right),\;\Phi \left(t^{last}\right)=0\;.$$

Then, one generates an exponentially distributed random number *u* and finds $t^{new}$ such that

$$\Phi (t^{new})=-\ln u\;.$$

This replaces Step 1 in the standard GDM above.

Then, in the GDM, which reaction occurs is decided according to a probability

$$\Pi_{i}\left(t^{new}\right)=\frac{\lambda_{i}\left(t^{new}\right)}{\Lambda (t^{new})}\;.$$

The procedure based on Equation (16) has been adopted in [51], where it was mentioned that finding τ from this equation might be time-consuming. This algorithm is described in [35] as Algorithm 2.

The exact approach above is discussed in [35,52]. In Section 2.2.4, we describe how the exact algorithm above can be efficiently implemented.

#### 2.2.3. Thinning Method

The thinning method (first suggested in [53]) provides an alternative implementation for exact sampling of event times under time-dependent rates. We do not go into details and refer to the book [47], Section 5.4.5, for a description of this method.

In the context of simulations of neural models, it is important to note that the algorithm is rather efficient if the rate λ(t) is an explicit function of time (it can even be another stochastic process); however, if λ depends on a dynamical variable obeying an ODE, then multiple integrations of Equation (17) are needed. For one HH neuron, the equation for *V* in (1), (2) is *linear* in variable *V*, and the coefficients of this linear equation are constant between the jumps. Thus, the solution can be written explicitly and used in the thinning method, as was implemented in [41]. However, this approach does not work for the ML model, where the voltage equation is nonlinear (cf. [36]).

#### 2.2.4. An Efficient Method for Stochastic Modeling of General PDMPs

Here, we outline the advanced efficient algorithm for simulating generic PDMPs that was recently proposed in [29]. We rewrite Equations (13), (14) and (17) as

(18) $$\begin{array}{cc} \frac{d\vec{X}}{dt} & =\vec{F}\left(\vec{X},\vec{Y},t\right)\;,\\ \frac{d\Phi }{dt} & =\Lambda (\vec{X},\vec{Y},t)\;, \end{array}$$

with initial condition $\vec{X}\left(t^{last}\right)$, $\Phi (t^{last})=0$. In (18), the discrete states $\vec{Y}$ are constants.

The trajectory of (18) should end at time $t^{new}$, at which Φ=Δ=−lnu, where *u* is sampled from a uniform distribution 0<u≤1. Finding the corresponding time is the most expensive part of the algorithm if the total rate Λ depends on time. If Λ is constant between discrete transitions, then $\Phi =\Lambda (t-t^{last})$ and the solution is trivial: $t^{new}=t^{last}+\Delta /\Lambda$.

We can consider Φ in (18) as independent variable and rewrite these equations (this transformation was first suggested by M. Henon in the context of deterministic dynamics [54]):

(19) $$\begin{array}{cc} \frac{d\vec{X}}{d\Phi } & =\frac{\vec{F}\left(\vec{X},\vec{Y},t\right)}{\Lambda (\vec{X},\vec{Y},t)}\;,\\ \frac{dt}{d\Phi } & =\frac{1}{\Lambda (\vec{X},\vec{Y},t)}\;. \end{array}$$

For the system in (19), the initial conditions at Φ=0 are $\vec{X}\left(t^{last}\right)$, $t^{last}$. These equations are integrated on the prescribed interval of the independent variable 0≤Φ≤Δ, which is a standard task for numerical solutions of ODEs.

For example, it is possible to use the standard fourth-order Runge–Kutta method with a constant step size Δ/Q, performing an integer number *Q* of integration steps. If one requires an integration step not larger than Δt, then one can choose Q=[Δ/Δt]+1, where [·] is the integer part of a real number. Alternatively, one can use a method with accuracy control (e.g., the Runge–Kutta–Dormand–Prince-45 method) and automatic step size adjustment. The solution of (19) yields the new values of the time $t^{new}=t\left(\Delta \right)$ and continuous variables $\vec{X}\left(t^{new}\right)=\vec{X}\left(\Delta \right)$.

After finding $\vec{X}\left(t^{new}\right)$, $t^{new}$, one completes the GDM by choosing the proper reaction according to the probabilities

(20) $$\Pi_{k}=\frac{\lambda_{k}\left(\vec{X}\left(t^{new}\right),\vec{Y},t^{new}\right)}{\sum_{k}\lambda_{k}\left(\vec{X}\left(t^{new}\right),\vec{Y},t^{new}\right)}\;.$$

Below, we use this algorithm in all simulations.

### 2.3. Characterization of the Regular Component by Virtue of the Wiener Order Parameter

Here, we present a method for quantifying the response of a stochastic neuron to periodic forcing, following [55]. Under periodic forcing, a regular component with the frequency of forcing appears in the stochastic voltage signal V(t). First, from the process V(t), calculate the autocovariance function (ACF):

(21) C(τ)=〈(V(t)−〈V〉)(V(t+τ)−〈V〉)〉.

In the autonomous stochastic case, this ACF decays to zero at large time lags τ. In contrast, with a periodic forcing the ACF has an initial decay, then is periodic in τ for large time lags. The average of $C^{2}\left(\tau \right)$ in this region of large time lags is the Wiener parameter:

(22) $$W=\frac{1}{\Theta_{2}-\Theta_{1}}\int_{\Theta_{1}}^{\Theta_{2}}\;d\tau \;C^{2}\left(\tau \right)\;.$$

This parameter measures the squared total mass of the point spectrum in the power spectrum of the process [56]. Because the ACF is already the squared voltage, a natural way to define the “amplitude” of the regular component is to take $W^{1/4}$. For small periodic forcing in the regime of linear response, this amplitude is proportional to the amplitude of the driving.

## 3. Results

### 3.1. Effect of EMFs on Firing Rates for Excitable Neurons

One condition for a neuron to be excitable is the existence of a unique stable steady state in the deterministic limit (infinite number of ion channels). To produce a spike, such a neuron needs an external input. Numerical simulations related to safety standards consider the periodic driving of a deterministic neuron model as described by the expression in (12) and determine a critical amplitude at which spikes appear [23,28,37,57]. The precise modeling of body-induced currents, i.e., the input of (12), is a highly non-trivial dosimetric task [58,59,60], particularly for EMF frequencies lower than a few MHz [61]. For stochastic neurons (finite number of channels), spontaneous spikes can appear without external input [15]. Below, we characterize the firing rate of the stochastic HH and ML models, with an emphasis on comparisons with deterministic calculations. We investigate these models due to their popularity in the neuroscience literature. Our analyses should serve as an indication of what happens in the FH model as the basis for the SENN protocol.

#### 3.1.1. HH Model

##### Autonomous Stochastic HH Model

**Figure 1:**
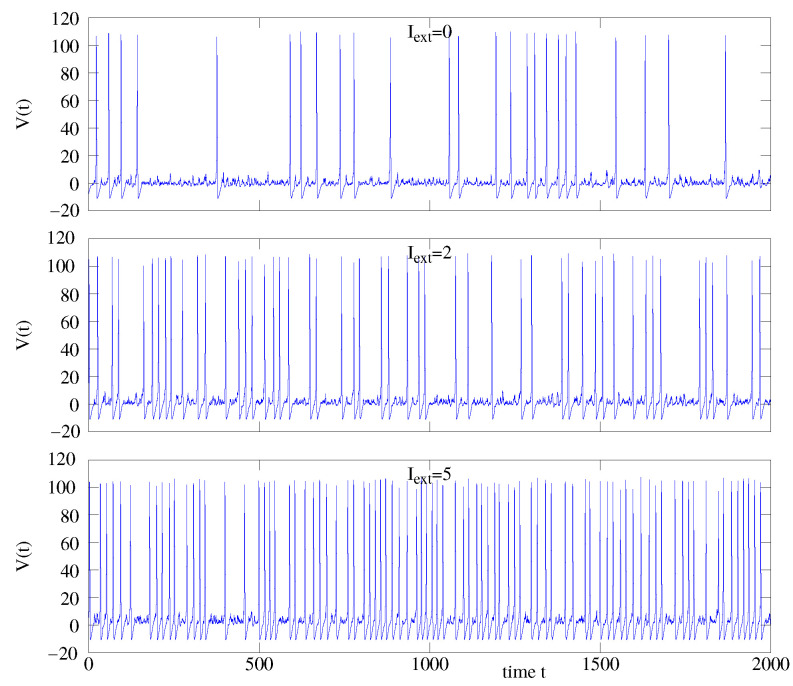
Stochastic simulations of the HH model (4)–(6) with M=6000 and N=1800 (these numbers were used in the pioneering simulations in [62] and in many subsequent studies). The deterministic model for all these parameters has a stable steady state; spikes are excited by ion channel noise (units: voltage in mV, time in ms).

We start with the HH model (4)–(6) and illustrate spontaneous spiking due to ion channel noise in Figure 1. The main bifurcation parameter is $I_{ext}$; for large values of $I_{ext}$, periodic spiking occurs. Correspondingly, excitation of a spike requires a smaller perturbation for $I_{ext}=5$ compared to $I_{ext}=0$, which results in a larger firing rate. According to the time series of V(t) in Figure 1, we choose the threshold V=80 as a criterion for spike occurrence.

**Figure 2:**
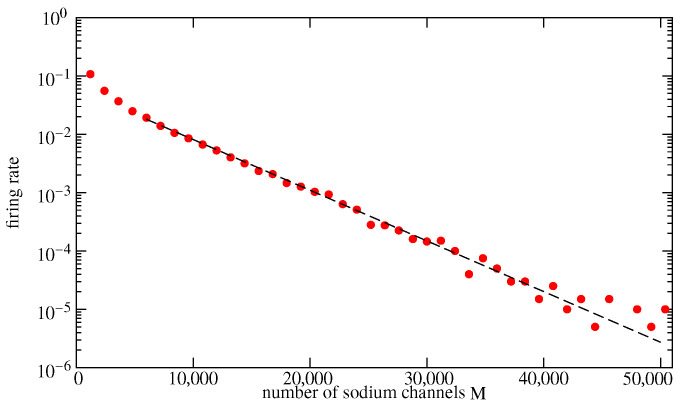
Firing rates (i.e., the number of spikes per time) for HH with channel noise vs. the number of Na channels (with a fixed ratio M/N=10/3) for $I_{ext}=0$. The total simulation time is $2\times 10^{5}$, which determines the lowest observed rate. The black dashed line fits the rate with the exponential $r\approx 0.06\exp[-2\times 10^{-4}N]$.

For a given value of $I_{ext}$ below the excitation threshold, the rate of spontaneous spike excitation depends on the effective noise intensity, which is inverse proportional to the number of channels [6,9,10,18,19,62,63]. The general Freidlin–Wentzel theory [64] predicts that for small noise up to a prefactor, the probability of excitation is exponentially small in noise intensity; thus, the firing rate is ∝exp[−aN], where *N* is the number of channels. This is illustrated in Figure 2.

##### HH Model: Spike Rates

**Figure 3:**
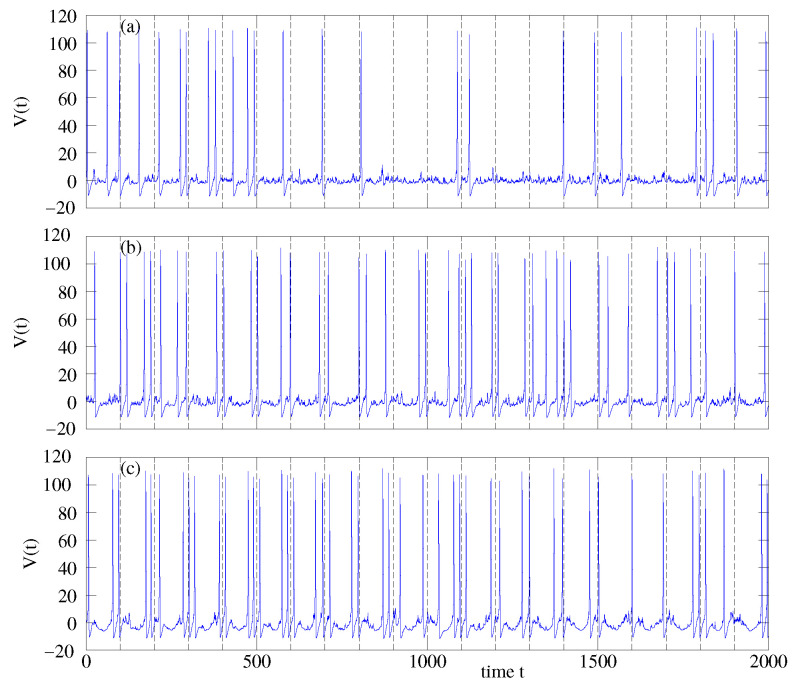
The same stochastic simulations of the HH model (4)–(6) as in upper panel of Figure 1 ($I_{ext}=0$) but with periodic driving. The driving frequency is f=10 Hz, meaning that the period is 100 ms (marked with vertical dashed lines on the panels). Panel (**a**) A=1; panel (**b**) A=2, panel (**c**) A=4 (units: voltage in mV, time in ms).

Here, we take an HH neuron in an excitable regime at $I_{ext}=0$ and look at how the number of generated spikes depends on the parameters of the forcing (amplitude and frequency) introduced according to (12). For an illustration of the spiking fields, Figure 3 presents three time series for the same period of forcing and different amplitudes. The corresponding autonomous case is the upper panel of Figure 1. For strong enough forcing, it can be seen that the spikes become concentrated at a certain phase of the driving force, although they still remain random.

**Figure 4:**
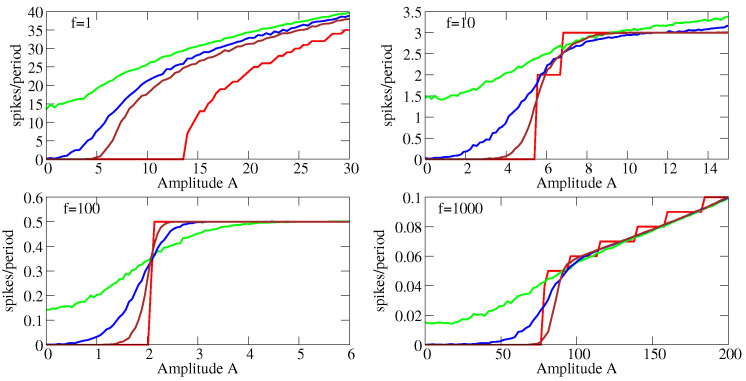
Average number of spikes per period vs. driving amplitude for different forcing frequencies (measured in Hz). Red line: deterministic simulation. Green line: numbers of channels M=6000, N=1800. Blue line: $M=3\times 10^{4}$, $N=9\times 10^{3}$. Brown line: $M=12\times 10^{4}$, $N=36\times 10^{3}$.

For statistical evaluation, we calculate the average number of spikes per period. Together with simulations using a finite number of ion channels, Figure 4 presents the results for the deterministic case (Equation (4)). In the deterministic case, there is a sharp transition as the amplitude increases, while in the stochastic case there is no such sharp transition. This is because spikes due to randomness can still appear even in the excitable state, albeit rarely. To illustrate this, we calculate spike rates at different frequencies and amplitudes of the forcing and compare deterministic results with stochastic simulations, with the results shown in Figure 4. Note that the absolute spike rates in spikes per second can be easily recovered from the spikes per period and length of the period.

This figure demonstrates that stochastic simulations definitely deviate from deterministic results in many cases. The graphs show that for the most typically used numbers of ion channels M=6000,N=1800 (these values are used in [18,19,62,63], and many cases consider even smaller systems), the spike rate (green line in Figure 4) is already relatively large at vanishing force. The number of spikes grows with the amplitude, but this dependence is not a “threshold-like” one. Therefore, we performed simulations with larger systems, keeping the ratio M/N=10/3 and increasing the number of ion channels by factors of 5 (blue line) and 20 (brown line). For such system sizes, the spiking probability is very small under vanishing forcing and grows monotonically with the forcing amplitude. For very small frequencies (see the panel for f=1 Hz), spiking appears significantly below the deterministic threshold (factor ≈2 for $M=12\times 10^{4}$). At higher frequencies and at the largest tested system size $M=12\times 10^{4}$, the threshold of spiking in the presence of ion channel noise is close to the deterministic case. Remarkably, the ion channel noise slightly suppresses the deterministic spiking rate for the largest frequency f=1 kHz. This comparison of deterministic and stochastic simulations of the spike excitation by periodic EMFs shows that, with the exception of ultra-low frequencies, the excitation threshold in the HH model with a large number of ion channels is only weakly sensitive to the stochasticity level.

##### HH Model: Periodic Component in the Spiking Train

**Figure 5:**
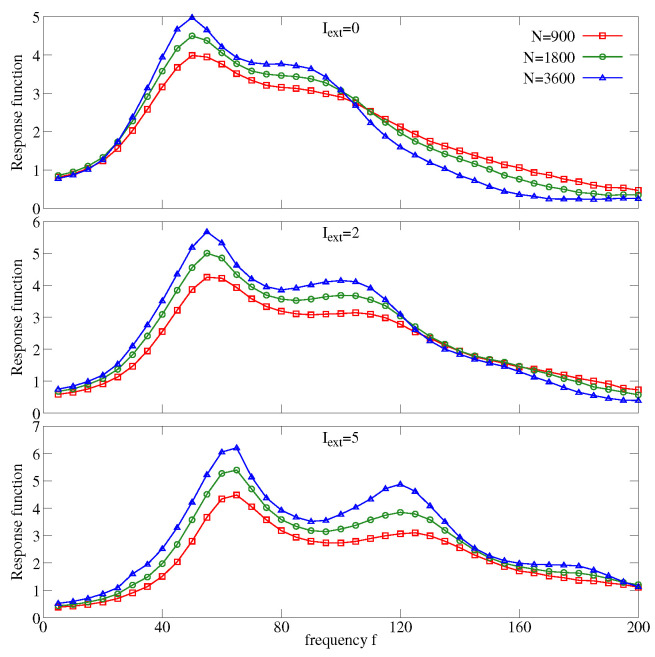
Response function $W^{1/4}/A$ for a driven stochastic HH model as a function of the frequency *f* (in Hz); the number of potassium channels *M* is given in the key legends, and the corresponding number of sodium channels is M=10N/3.

As has been demonstrated above, for the typical system sizes adopted in previous stochastic simulations of the HH model, the average spike rate depends only weakly on the forcing amplitude; however, even weak periodic forcing induces some regularity in the spikes. We quantify this regularity using the Wiener order parameter *W*, as described in Section 2.3. A linear response can be expected at small forcing amplitudes, where $W^{1/4}$ is proportional to *A*; thus, we calculate the frequency-dependent “response function” as $W^{1/4}/A$ for A=2. This function is presented in Figure 5 for several values of the parameter $I_{ext}$ and for several system sizes.

The major observation is that the response has a resonance shape, with a maximum around 50–60 Hz. This maximum is slightly more pronounced for smaller channel noise (larger sizes), but the effect is not strong: increasing the number of channels by a factor of 4 leads to 20% increase of the maximal response. Interestingly, for $I_{ext}=5$ there are two maxima, at f≈60 Hz and f≈120 Hz.

#### 3.1.2. ML Model

On the qualitative level, the dynamics of the ML model under channel noise are similar to that of the HH model. However, because the deterministic case is two-dimensional, some effects are easier to interpret and to approach analytically. Furthermore, the simulations are faster because only one type of random channels is present, and as such can be more easily extended to studies of networks of coupled neurons.

##### Autonomous Stochastic ML Model

**Figure 6:**
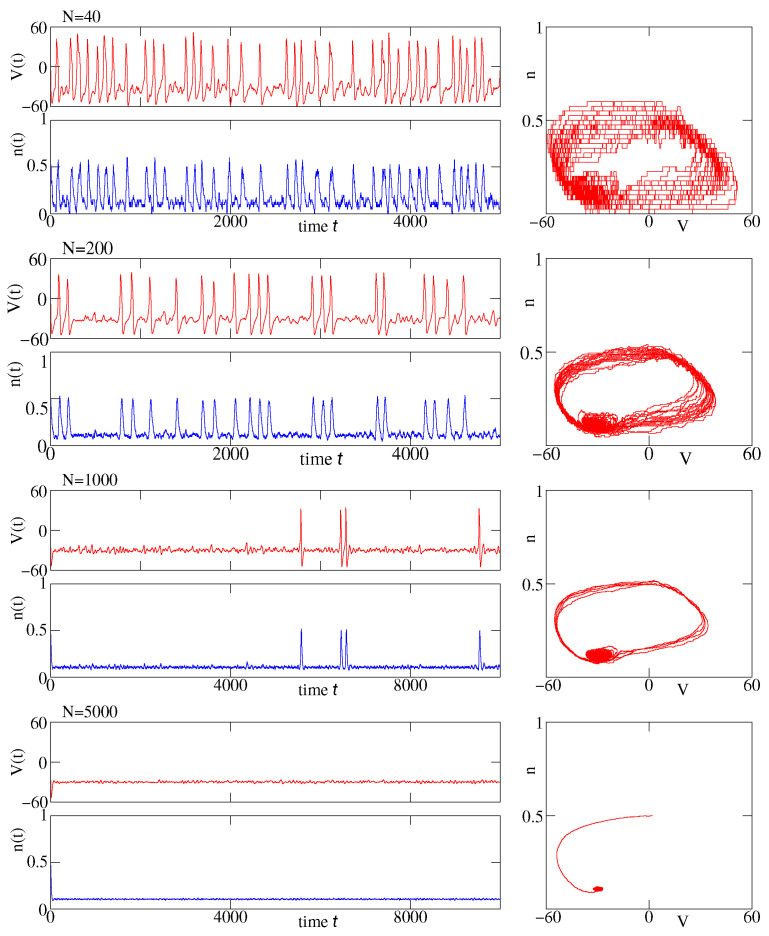
Regimes in the excitable case of the ML model for $I_{ext}=80$ for different number of ion channels (variable *V* (red lines) is in mV, variable *n* (blue lines) is dimensionless, and time is in ms). The right panels show the “phase portraits” in the (V,n) plane. For n=40, the discreteness is clearly visible in this panel.

Figure 6 shows regimes in the ML system for $I_{ext}=80$, where in the deterministic limit there is a stable steady state (a transition to periodic spiking in the deterministic case occurs at $I_{ext}\approx 88.5$). It can be seen that spikes become rare for large *N*, and practically disappear (on the time scale presented) for N=5000. According to Figure 6, a proper threshold for the detection of a spike is V=0.

**Figure 7:**
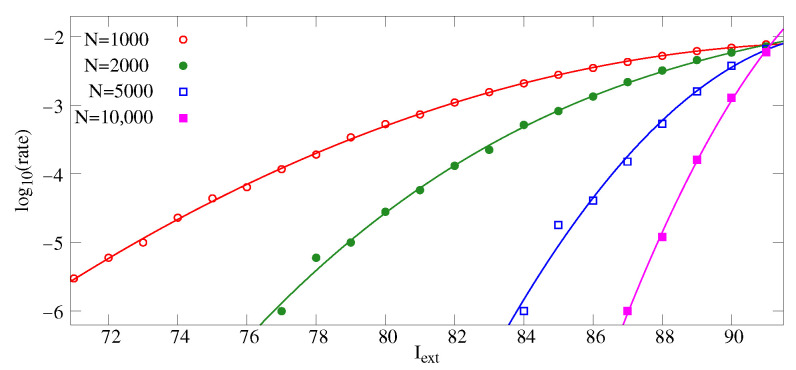
The markers show numerically obtained rates via the Markov model simulations, while the lines show polynomial fits (as Appendix A). Note that the natural quantity to fit here is the logarithm of the rate, since the rate is exponentially small for large system sizes.

Dependence of the spike rate on the parameter $I_{ext}$ is illustrated in Figure 7, where the numerically determined spike rates for different values of the number of channels are shown. We mention here that although the ML model is relatively simple, there are no analytical results about the spike rates (in contradistinction to even simpler models like one-dimensional integrate-and-fire neuron under the influence of white Gaussian noise, where such analytical results are possible [65]). However, as we will show below, it is possible to relate the static spike rates (i.e., the rates of the autonomous system) of Figure 7 to rates in the presence of slow periodic forcing (again, analytical results are available here for simple one-dimensional cases only [17,66,67]). To this end, having safety standards in mind, we determine numerical fits (solid lines in Figure 7) to the rates in the interval $I_{ext}<91$, where these rates exhibit non-trivial behavior. We use these functions to estimate the spike rates for a periodically forced ML neuron in Section ML Model: Spike Rates below. We believe that for higher-dimensional models used in the SENN protocol, such as HH or FH, this is the most direct way to obtain approximate rate functions. We are not aware of any analytical results thus far.

##### ML Model: Spike Rates

**Figure 8:**
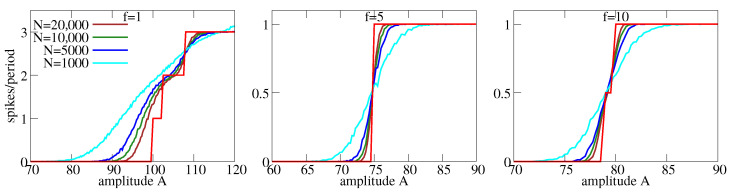
Spike rates (average number of spikes per period) vs. amplitude of the forcing *A* for different frequencies *f* (in Hz) and different numbers of channels *N* for the ML model. Here, we set $I_{ext}=0$; deterministic calculations are additionally shown in red. For the corresponding results for the HH model, see Figure 4.

Here, we add a periodic force according to (12) and calculate the spike rate. Figure 8 shows spikes per period vs. the amplitude of forcing for several periods of forcing (cf. similar data for the HH neuron in Figure 4). The number of spikes is calculated as the number of events at which the level V=0 is crossed. Relatively large deviations from the deterministic limit can be seen for N=1000, with smaller deviations apparent for larger values of *N*. Similarly to the properties of the HH model (Figure 4), the correspondence between the deterministic threshold and thresholds in the presence of channel noise is better for larger frequencies.

It is desirable to have a computational procedure for estimating the spike rate in dependence on the external signal $I_{ext}\left(t\right)$ without performing the full stochastic simulation. Motivated by this, we present an attempt to relate the rates at periodic forcing presented in Figure 8 to the static rates presented in Figure 7. For each value of *N*, the static rates depend on $I_{ext}$: $R(I_{ext})$. For a periodic forcing, the external current is $I_{ext}=A\cos\left(2\pi t/T\right)$. We adopt an adiabatic approximation, namely, that the time-dependent rate can be calculated just as $R\left(I_{ext}\left(t\right)\right)=R\left(A\cos\left(2\pi t/T\right)\right)$. Then, the average number of spikes per period of the forcing can be calculated as

(23) $$n\approx \int_{0}^{T}R\left(A\cos\left(2\pi t/T\right)\right)\;dt\;.$$

**Figure 9:**
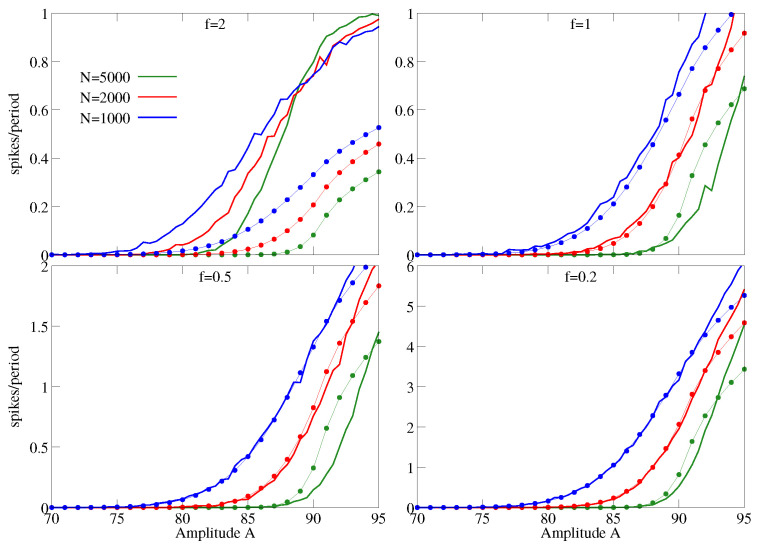
Comparison of numerically observed numbers of spikes per period (bold lines) with predictions of the formula in (23) (lines with filled circle markers of the corresponding color).

This expression should be applied to regimes prior to the deterministic transition to spiking, i.e., to small values of the amplitude *A*. Practically, we use the fits of the $R(I_{ext})$ dependencies presented in the expressions in (A1) when calculating the integral in (23). The results are presented in Figure 9.

It can be seen from Figure 9 that this approach works rather well for very low forcing frequencies (f=0.2), and is less precise for larger frequencies (f=1 and f=0.5) as well as for a relatively large number of channels (N=5000, green curve). For f=2, the correspondence is already poor.

##### ML Model: Periodic Component in the Spiking Train

**Figure 10:**
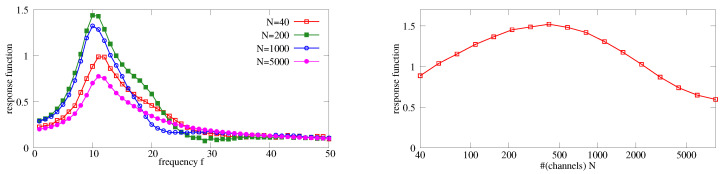
Response function of the ML model calculated for $I_{ext}=80$ (this value of the constant external current corresponds to a non-oscillatory steady state) and A=5. Left panel: Frequency dependence at different numbers of ion channels. Right panel: Dependence on the number of channels for fixed driving frequency f=10 Hz.

Here, we report on the calculations of the periodic component in the spike train induced by periodic forcing (12). The response function, defined as $W^{1/4}/A$ (where *W* is the Wiener order parameter (22) and *A* is the forcing amplitude), is shown in Figure 10. This figure should be compared with the corresponding result for the HH model in Figure 5. For the ML model, we observe a resonant response with a single maximum close to f≈10 Hz. An interesting feature is that the response at this maximum is non-monotonic in the number of channels, peaking around N=500. This is a manifestation of the stochastic resonance phenomenon, first reported for ion channel noise in [68,69].

**Figure 11:**
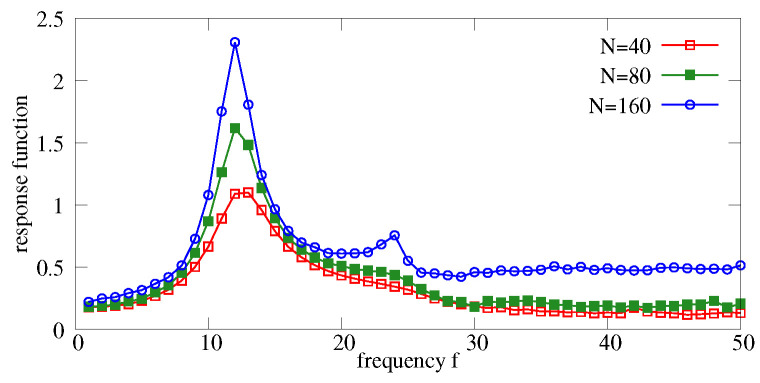
Response function of the ML model calculated for $I_{ext}=100$ (this value of the constant external current corresponds to the oscillatory regime in the deterministic limit) and A=5.

Above, we focus on the case where the neuron is in the excitable state, i.e., where the parameter $I_{ext}$ is below the threshold of spiking activity in the deterministic limit of an infinite number of channels. Of course, the same methods can be applied to the spiking neuron as well. In the deterministic limit, features such as synchronization and the onset of chaos can be observed, while stochasticity dominates for a relatively small number of channels. For such a situation, the response to a relatively small periodic force is approximately the same as in the excitable state. We illustrate this with Figure 11, which differs from Figure 10 only in the value of parameter $I_{ext}=100$, which is beyond the spiking threshold $I_{ext}\approx 88$. It can be seen that the resonant response at f≈10 is more pronounced, and already peaks with a relatively small number of channels N=160 at which deterministic features start to dominate. For the ML model, we observe just one dominant peak in the response function (Figure 10 and Figure 11), while the HH model demonstrates two peaks for some values of parameters (Figure 5). A possible interpretation of this is that the deterministic ML model is two-dimensional, i.e., it has just one oscillating mode, while the four-dimensional HH model allows for different oscillating modes.

## 4. Discussion

In this paper, we have explored the effect of an external periodic signal on a neuron in the presence of ion channel stochasticity. Two paradigmatic models have been studied, namely, the Hodgkin–Huxley and Morris–Lecar systems. Random openings and closings of the ion channels in these models can be represented as piecewise-deterministic Markov processes. An efficient numerical method applicable both for autonomous and periodically driven neurons has been implemented in all numerical simulations. We focus on the properties of neural activity relevant to the safety assessment of electromagnetic field effects. Currently, safety regulation simulations rely on deterministic models, and extending them to incorporate ion channel stochasticity is an important area for future investigation. Our study focuses on exploring the dynamics of a single neuron under the assumption of a given driving current. For the purpose of determining threshold values of external electromagnetic fields, it is necessary to combine this with simulations of the frequency-dependent transformation of the fields in the body and neural tissue and then calculate the corresponding induced transmembrane currents, as done in, e.g., SENN [20,21,22,26,28] and the modeling of transcranial electrical stimulation [70] (with potential usage of computational environments [71,72]). Especially for frequencies below several MHz, this is currently an active field of research [60,61].

The main difference between stochastic and deterministic models is that in the latter a sharp transition to spiking activity in an excitable neuron occurs, whereas in the former this transition is smeared due to the spontaneous appearance of random spikes. We characterize spike rates in the presence of an external field and ion channel noise across different parameters of the periodic forcing (frequency and amplitude) and different numbers of channels (system sizes). At low channel noise (large numbers of ion channels), significant spiking activity below the deterministic amplitude threshold is observed at low frequencies, whereas at high frequencies the effect of channel noise on the threshold is small. Hence, deterministic models seem to overestimate firing thresholds for low frequencies. A quantitative result for the Frankenhäuser–Huxley model is planned for future work. For a relatively small system with a small number of ion channels, spontaneous stochastic activity is already strong, and periodic forcing has only a small contribution to its level. However, this contribution is regular, and as such can be characterized by a periodic component appearing in the voltage signal. We quantify this component using the Wiener order parameter, which can be readily computed from the signal’s autocorrelation function. For small forcing amplitudes, the periodic component of the spiking activity is proportional to the forcing amplitude; its frequency dependence is the response function. For both HH and ML models, this response function has a resonance-like shape for the chosen set of parameters, with the highest sensitivity at 50 Hz for HH and 10 Hz for ML. Interestingly, in experimental studies of the magnetophosphene effect [73,74] (visual sensation induced by periodic magnetic fields), the threshold is the lowest in the range of 10–30 Hz [73]; under electrical stimulation [75], the maximal phosphene response is in the range of 10–20 Hz.

The presence of a periodic component can potentially influence information processing in neural networks, posing another important issue for safety considerations. However, in order to evaluate this it would be necessary to extend the present study to networks of coupled neurons (cf. [76]), with thorough comparison to experiment.

Next, we discuss the relation to other numerical and analytical approaches. In modeling PDMPs, one often approximates the ion channel’s opening and closing rates with constants, meaning that the standard Gillespie simulation algorithm is applicable. This can be justified only for a very large number of channels, whereas the method described in this paper works for any number of channels. One can implement an exact Monte Carlo simulation of channel noise using an iterative numerical approach, as in [35], but this is less efficient than the presented method.

For a large number of channels, the diffusion approximation, which reduces the PDMP to a stochastic differential equation (SDE), has been shown to be valid asymptotically [6,12]. This enables stochastic simulations based on numerical methods for SDEs. However, additional issues appear; in particular, the portion of open ion channels in the SDE formulation is not restricted to the interval between zero and one. Additionally, the accuracy of simulations depends only weakly on the time step; whereas in the presented method, which uses Runge–Kutta integration, this dependence is strong.

Analytical approaches to the calculation of spike rates are typically formulated as general asymptotic expressions, where exponential dependence on the number of channels dominates [77,78]. Formulae that are also valid for non-exponentially small spiking are available only for the simplest one-dimensional integrate-and-fire models [65]. In this investigation, we have tested a phenomenological adiabatic approximation to the effect of periodic forcing on the spike rate. For low frequencies of the external force, we use numerically-obtained static spike rates to estimate the spike rate in the presence of forcing. However, this approach only works for very low frequencies (less than 1 Hz).

The presented method for modeling ion channel randomness is not limited to periodic forcing; it applies to any force that can be represented as a piecewise-smooth function of time. Because the method is based on integrating a system of ordinary differential equations, an analytical representation of the force is required for Runge–Kutta-type integration to be applicable. For example, a force in the form of a sequence of modulated pulses [79] is allowed; however, the method cannot be applied if the force is a random function of time.

Finally, we remark that there are other fields of research where the effect of periodic fields on neurons is important. For example, electrical and magnetic forcing is adopted in brain stimulation, including deep brain stimulation and transcranial brain stimulation, [70,80,81], as well as in high-frequency microwave stimulation [82,83]. Mechanical and acoustic forces [84] can also be explored using the same methodology.

## 5. Conclusions

In summary, this paper applies the exact method for simulation of piecewise-deterministic Markov processes to model Hodgkin–Huxley and Morris–Lecar neuron dynamical systems in the presence of ion channel stochasticity and periodic external driving. The effect of stochasticity is mostly pronounced at low frequencies, where it significantly reduces the value of the driving amplitude at which spiking appears. We discuss a semi-analytic adiabatic approach that allows for calculation of the spiking rate at periodic driving based on the static rate, and demonstrate that it works for frequencies below 1 Hz. Furthermore, we characterize the regular component in the spiking via the Wiener order parameter and demonstrate that the response typically has a peak in the beta range. The proposed method can be incorporated in packages such as the SENN package that are used for simulating neural dynamics in external electromagnetic fields, but has a limitation in that only deterministic driving protocols are allowed.

## Data Availability

All the data in this paper were obtained using standard methods and the described algorithms. The raw data supporting the conclusions of this article will be made available by the authors on request.

## Abbreviations

The following abbreviations are used in this manuscript:

EMF	Electromagnetic field
HH	Hodgkin–Huxley model
ML	Morris–Lecar model
FH	Frankenhaeuser–Huxley model
SENN	Spatially Extended Nonlinear Node model
PDMP	Piecewise-Deterministic Markov Process

## Appendix A. Fitted Spike Rates for the ML Model

The fitted functions in Figure 7 are as follows:

(A1) $$\begin{array}{c} f_{N=1000}\left(x\right)=-3.1256+0.17\left(x-81\right)-0.007038{\left(x-81\right)}^{2}+8.19\cdot 10^{-6}{\left(x-81\right)}^{3}\;71\leq x\leq 91\;,\\ f_{N=2000}\left(x\right)=-3.582+0.281\left(x-83\right)-0.015{\left(x-83\right)}^{2}+0.000349{\left(x-83\right)}^{3}\;77\leq x\leq 9\;,\\ f_{N=5000}\left(x\right)=-3.7417+0.56396\left(x-87\right)-0.044245{\left(x-87\right)}^{2}\;84\leq x\leq 9\;,\\ f_{N=10000}\left(x\right)=-3.8164+0.9579\left(x-89\right)-0.075{\left(x-89\right)}^{2}\;87\leq x\leq 91\;. \end{array}$$
